# Supplementary material for: Identification of photoperiod-induced specific miRNAs in the adrenal glands of Sunite sheep (Ovis aries)
Source: Front Vet Sci. 2022 Jul 22;9:888207. doi: 10.3389/fvets.2022.888207 (PMC9354845; doi:10.3389/fvets.2022.888207)
Supplement: Supplementary file 1 [file Data_Sheet_1.ZIP › Supplementary file/miRDeep2 file.zip!/miRDeep2 file/Know_Structure/SP42A2/oar-mir-380.pdf]

miRBase precursor : oar-mir-380
Total read count : 2974
oar-miR-380-5p read count : 33
oar-miR-380-3p read count : 2941
remaining reads : 0

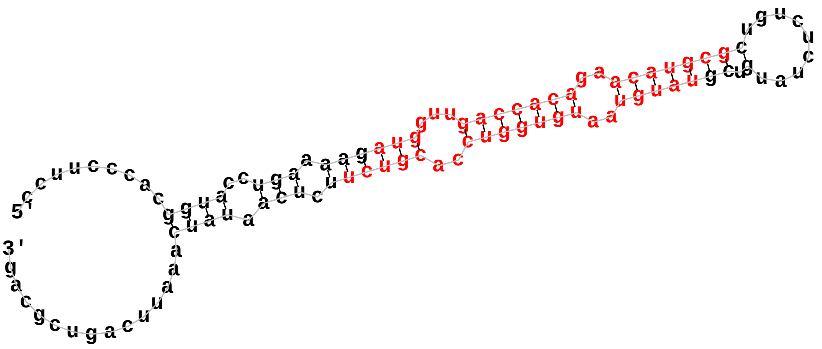

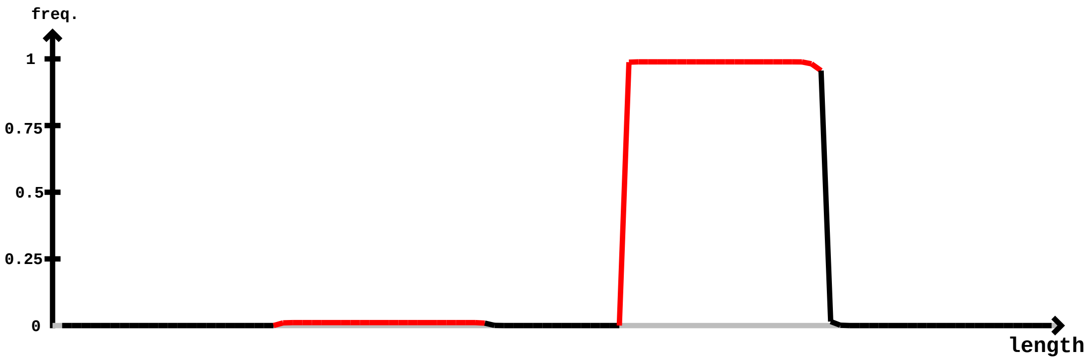

oar-miR-380-5p

oar-miR-380-3p

```
5'- ccuucccacgguaccugaaaagaugguugaccacagaacaugcgcugucucuaugucguauguaaugugguccacgucuucucaauaucaaauucagucgcag   -3'    exp
    .........((((..(((.((((((...(((((((..((((((((.........).)))))))..)))))))..)))))).))).)))).............     reads      mm         sample
    ......................augguugaccacagaacaugc............................................................     6          0          seq
    ......................augguugaccacagaacaugcg...........................................................     22         0          seq
    ......................augguugaccacagaacaugcgc..........................................................     2          0          seq
    .......................ugguugaccacagaacaugcg...........................................................     3          0          seq
    ..........................................................uauguaaugugguccacg...........................     1          0          seq
    ..........................................................uauguaaugugguccacgu..........................     21         0          seq
    ..........................................................uauguaaugugguccacguc.........................     76         0          seq
    ..........................................................uauguaaugugguccacgucu........................     2794       0          seq
    ..........................................................uauguaaugugguccacgucuu.......................     41         0          seq
    ..........................................................uauguaaugugguccacgucuuc......................     4          0          seq
    ...........................................................auguaaugugguccacgucu........................     4          0          seq
```
